# Role of Polymorphisms of Inducible Nitric Oxide Synthase and Endothelial Nitric Oxide Synthase in Idiopathic Environmental Intolerances

**DOI:** 10.1155/2015/245308

**Published:** 2015-03-24

**Authors:** Chiara De Luca, Agnese Gugliandolo, Carlo Calabrò, Monica Currò, Riccardo Ientile, Desanka Raskovic, Ludmila Korkina, Daniela Caccamo

**Affiliations:** ^1^Centre of Innovative Biotechnological Investigations (Cibi-Nanolab), 197 Vernadskogo Prospekt, Moscow 119571, Russia; ^2^Active Longevity Clinic “Institut Krasoty na Arbate”, 8 Maly Nikolopeskovsky lane, Moscow 119002, Russia; ^3^Department of Biomedical Sciences and Morpho-Functional Imaging, Polyclinic University of Messina, 98125 Messina, Italy; ^4^2nd Dermatology Division, Dermatology Institute (IDI IRCCS), Via dei Monti di Creta 104, 00167 Rome, Italy

## Abstract

Oxidative stress and inflammation play a pathogenetic role in idiopathic environmental intolerances (IEI), namely, multiple chemical sensitivity (MCS), fibromyalgia (FM), and chronic fatigue syndrome (CFS). Given the reported association of nitric oxide synthase (NOS) gene polymorphisms with inflammatory disorders, we aimed to investigate the distribution of NOS2A −2.5 kb (CCTTT)_*n*_ as well as Ser608Leu and NOS3 −786T>C variants and their correlation with nitrite/nitrate levels, in a study cohort including 170 MCS, 108 suspected MCS (SMCS), 89 FM/CFS, and 196 healthy subjects. Patients and controls had similar distributions of NOS2A Ser608Leu and NOS3 −786T>C polymorphisms. Interestingly, the NOS3 −786TT genotype was associated with increased nitrite/nitrate levels only in IEI patients. We also found that the NOS2A −2.5 kb (CCTTT)_11_ allele represents a genetic determinant for FM/CFS, and the (CCTTT)_16_ allele discriminates MCS from SMCS patients. Instead, the (CCTTT)_8_ allele reduces by three-, six-, and tenfold, respectively, the risk for MCS, SMCS, and FM/CFS. Moreover, a short number of (CCTTT) repeats is associated with higher concentrations of nitrites/nitrates. Here, we first demonstrate that NOS3 −786T>C variant affects nitrite/nitrate levels in IEI patients and that screening for NOS2A −2.5 kb (CCTTT)_*n*_ polymorphism may be useful for differential diagnosis of various IEI.

## 1. Introduction

The number of people affected by idiopathic environmental intolerances (IEI), now also more appropriately called environmental “sensitivity-related illnesses” (SRI) with difficult diagnosis, is constantly growing worldwide. IEI include the most frequent multiple chemical sensitivity (MCS), chronic fatigue syndrome (CFS), fibromyalgia (FM), electromagnetic hypersensitivity (EHS), amalgam disease, and others [1]. The clinical IEI features include multiorgan manifestations in respiratory, nervous, cardiological, endocrine, cutaneous, and gastrointestinal systems, often without classic allergologic and/or immunologic markers [1–3]. In many cases, disease onset appears to be preceded by a short-term stress, most commonly a chemical overexposure in the case of MCS, infections in CFS, and physical trauma in FM, and then followed by a chronic condition that typically lasts for years or decades (for recent review see [2, 4, 5]).

The inherited or acquired impaired metabolism of xenobiotics has been postulated as a molecular basis for IEI pathogenesis. It has recently been demonstrated that some genetic variants of drug metabolizing and detoxifying enzymes, such as cytochrome P450 reductase (CYP), glutathione-S-transferase (GST), N-acetyl-transferase (NAT), and superoxide dismutase (SOD2), may be considered genetic determinants of IEI risk [2, 6–9].

These genetic polymorphisms seem to be connected to the loss of efficiency of detoxification systems, disturbances of free radical/antioxidant homeostasis, and increased production of inflammatory cytokines [1, 10, 11]. The overlapping symptoms among various IEI have prompted several investigators to hypothesize that they may share a common pathogenetic feature, and the most suitable one proposed so far is the increased nitric oxide/peroxynitrite levels. In fact, short-term stressors are able to increase systemic nitric oxide production [12, 13], which in the reaction with superoxide forms the potent oxidant peroxynitrite. Peroxynitrite, in turn, can act through six different positive feedback loop mechanisms to increase the levels of nitric oxide and its other precursor, superoxide anion, to form more peroxynitrite in a vicious cycle [14, 15].

Interestingly, it has recently been reported that gene variants of NOS2 are associated with alteration of NO levels in inflammatory bowel disorders, asthma, atopy, and migraine [16–20], all of which are comorbidities shared by IEI. Furthermore, circulating NO levels may also be affected by some variants of NOS3 gene [21].

This clinical case-control study aimed at investigating the association, if any, of the promoter pentanucleotide microsatellite −2.5 kb (CCTTT)_*n*_ and the Ser608Leu polymorphisms in NOS2 gene and the −786T>C polymorphism in NOS3 promoter with different IEI, such as MCS, suspected MCS (SMCS), FM, and CFS. We also attempted to find out a correlation between the indicated polymorphisms and serum levels of nitrite/nitrate.

## 2. Materials and Methods

### 2.1. Study Cohorts

Three hundred and sixty-seven Italian patients, who presented with symptoms compatible with IEI, were enrolled in this study at Department of Medical Pathophysiology, University of Rome “La Sapienza,” Policlinico Umberto I, and at Istituto Dermopatico dell'Immacolata (IDI IRCCS) in Rome, Italy (Ethical Committee Board Approval, Istituto Dermopatico dell'Immacolata, IDI IRCCS, Rome, Italy, n.121/CE/2008).

Diagnosis was set based on the medically assessed results of the modified QEESI (quick environmental exposure and sensitivity inventory) scoring [22]. QEESI is a validated self-administered questionnaire developed as a screening tool for patients with IEI. It is based on five different scales of assessment: symptoms severity, chemical triggers, other triggers, life impact, and finally a masking index to ongoing exposures [22]. A modified QEESI score of 10 common environmental exposures and 10 major symptoms enabled the differential diagnosis of MCS and SMCS (suspected MCS): full diagnosis (20 ≤ score ≤ 30) or strongly suspected diagnosis, that is, subjects fulfilling diagnostic criteria only partially (10 ≤ score ≤ 20) or subjects excluded from enrollment (0 ≤ score ≤ 10) [10].

One hundred and seventy of the recruited subjects were consecutive patients with MCS (49M/121F; 49 ± 11 years), 108 were consecutive patients with SMCS (90F/18M; 49 ± 12 years), who partly corresponded to the above reported diagnostic criteria, and 89 were consecutive patients (67F/22M; 47 ± 10 years) presenting with either fibromyalgia or chronic fatigue syndrome (FM/CFS).

One hundred and ninety-six healthy Italian subjects (M = 59, F = 137; 45.5 ± 9 years) were selected as controls among healthy staff members of participant institutions, Istituto Dermopatico dell'Immacolata (IDI IRCCS) in Rome and University of Messina, according to the established criteria as follows: (i) an absence of any clinically diagnosed disease, in particular allergic or immunologic disturbances, (ii) no drug or nutraceutical supplement since at least six weeks, at the time of blood sampling, and (iii) whole blood total production of reactive oxygen and nitrogen species (ROS/RNS) below 650 cps/L, as determined by luminol-dependent chemiluminescent response to phorbol 12-myristate 13-acetate [23] (subject recruitment under study protocol approved by Istituto Dermopatico dell'Immacolata—IDI IRCCS, Rome, Italy—Ethical Committee, n.52/CE/2010).

Nonsmokers in the patients' group were 81.4%, smokers 11.3%, and patients with undetermined smoking habits 7.3%. Nonsmokers in the control group were 85.2%. No alcohol or drug abusers were present among patients or controls.

Blood samples were drawn after all subjects provided written informed consent. The study was carried out in accordance with the Declaration of Helsinki (1964), and the study protocol was approved by the local Ethics Committee.

### 2.2. Genotyping for Single Nucleotide Polymorphisms NOS2A Ser608Leu and NOS3 −786T>C

Genomic DNA was isolated from peripheral leukocytes by PureGene DNA Purification System kit (Gentra Qiagen, Milan, Italy). Genotyping for the single nucleotide polymorphisms (SNPs) NOS2A Ser608Leu (C2087T, rs2297518) and NOS3 −786T>C (rs2070744) was performed by real-time PCR allelic discrimination, using TaqMan-Based Genotyping Assays (Applied Biosystems; assay ID: C_11889257_10; C_15903863_10) available from Life Technologies (Monza, Italy) in a 96-well plate on a 7900HT Fast Real-Time PCR System (Applied Biosystems, Foster City, CA, USA). The reactions were carried out in a final volume of 20 *μ*L containing 1x TaqMan Genotyping Master Mix, 1x TaqMan-based specific assay, and 10 ng genomic DNA, using thermal cycling conditions suggested by manufacturer's protocols.

### 2.3. Genotyping for NOS2A Pentanucleotide Microsatellite −2.5 kb (CCTTT)

Genotyping for the pentanucleotide microsatellite −2.5 kb (CCTTT)_*n*_ in NOS2A promoter was carried out by DNA direct sequencing. PCR reactions were carried out in a final volume of 50 *μ*L, containing 100 ng of genomic DNA, 1x PCR buffer, 1.7 mM MgCl_2_, 0.2 mM dNTP, 0.2 *μ*M primers, and 1 U EuroTaq DNA polymerase (EuroClone, Milan, Italy), in One Personal thermocycler (Celbio, Milan, Italy), using the following thermal cycling conditions: initial denaturation at 95°C for 10′, then 40 cycles of 95°C for 20′′, 56°C for 20′′, 72°C for 20′′, and a final extension step at 72°C for 7′. PCR primer sequences were the same used by Holla and coworkers [16].

PCR products were purified and the sequencing reaction was performed with BigDye Terminator v1.1 Cycle Sequencing kit (Applied Biosystems, Applera Corp., Milan, Italy). The reaction was carried out in a final volume of 20 *μ*L, containing 30 ng of purified PCR product, 1.6 pmol of forward primer, 2.5x ready reaction mix, 5x sequencing buffer, and DNAse/RNAse free water, in a thermocycler Hybaid ThermoSprint (Celbio, Milan, Italy). The thermal cycling conditions were 96°C for 3′, then 28 cycles of 10′′ at 96°C, 10′′ at 50°C, 4′ at 60°C, and finally 5′ at 4°C. The sequencing products were purified and analysed with ABI PRISM 310 Genetic Analyzer (Applied Biosystems, Applera Corp., Milan, Italy).

### 2.4. Statistical Analysis

To compare NOS2A and NOS3 allele and genotype frequencies in patients and controls, as well as deviations of genotype distributions from Hardy-Weinberg equilibrium, Fisher's exact test was used.

Statistical analysis was performed using the GraphPad Prism 4 software (San Diego, CA, USA).

The linkage disequilibrium extent between the two NOS2 polymorphisms was estimated by Haploview v4.2, by collecting the (CCTTT)_*n*_ alleles in two categories according to the number of repeats; that is, the alleles with <12 repeats were designated as s (short) and alleles with ≥12 repeats as l (long) alleles.

Statistical analysis of nitrite/nitrate variations in patients and controls was performed using STATISTICA 7.0 program (StatSoft Inc., Tulsa, OK, USA). Nitrite/nitrate plasma concentration values were presented as median, lower, and upper quartiles, minimum and maximum. Nitrite/nitrate plasma levels in either patients or controls having different NOS2A and NOS3 genotypes were analysed by one-way ANOVA. The differences in nitrite/nitrate plasma levels among the subgroups of patients and controls were analysed by two-way ANOVA. If necessary, *P* values were adjusted for multiple comparisons using the Bonferroni adjustment.

A *P* value of 0.05 or lower was regarded as statistically significant.

## 3. Results

### 3.1. Allele and Genotype Frequencies of NOS2A and NOS3 SNPs

Genotype and allele frequencies of NOS2A and NOS3 SNPs examined in IEI patients (MCS, SMCS, and FM/CFS) and controls are shown in Table 1. The genotype frequencies did not deviate from the expected value by Hardy-Weinberg equilibrium.

Genotyping for the SNP C2087T (Ser608Leu) in NOS2A showed that allele frequencies in patients were not statistically significant different in comparison with controls. The wild-type allele C was the most frequent in all groups and almost entirely represented by the homozygous genotype.

The CC2087 genotype frequency was higher in FM/CFS group than in all the other groups, and it tended to a statistically significant difference only in comparison with SMCS group (*P* = 0.08) that showed the lowest CC frequency.

The frequency of the homozygous genotype TT2087 was higher in patients than in controls, but these differences were not statistically significant. The heterozygous genotype was more represented in controls than in patients, except for SMCS patients. The FM/CFS group showed the lowest CT genotype frequency (Table 1).

The analysis of the distribution of NOS3 gene variant −786T>C showed that allele and genotype frequencies were not significantly different between the groups. The T wild-type allele was more frequent than the C mutant one. The heterozygous genotype was the most represented in all groups.

### 3.2. Allele and Genotype Frequencies of the Pentanucleotide Microsatellite −2.5 kb (CCTTT)_*n*_ in NOS2A Promoter

Genotyping results showed that the NOS2A −2.5 kb (CCTTT)_*n*_ repeat variant was present with nine different alleles (ranging 8–16 repeats, 176–216 bp) both in patients and controls, with an unimodal distribution having a peak at the (CCTTT)_12_ allele that was the most frequent in all examined groups (Table 2). The (CCTTT)_7_ and (CCTTT)_17_ alleles were not found in our study cohort.

The estimation of linkage disequilibrium for NOS2A C2087T and −2.5 kb (CCTTT)_*n*_ alleles showed that NOS2A C2087T is not in linkage disequilibrium with the microsatellite s/l allelic classification (*D*′ = 0.112, LOD = 0.24, *r* ^2^ = 0.003).

The (CCTTT)_8_ allele was more frequent in controls than in patients, showing a prevalence three-fourfold higher in controls than in MCS and SMCS patients. This difference was highly significant in comparison with MCS patients (*P* < 0.01) and significant in comparison with SMCS patients (*P* < 0.05); this allele was not observed among FM/CFS patients (Table 2). Notably, the presence of the (CCTTT)_8_ allele was associated with a decreased risk of IEI, since it reduced by threefold the risk for MCS (OR = 0.3; 95% C.I. = 0.12–0.77), by sixfold the risk for SMCS (OR = 0.16; 95% C.I. = 0.021–1.24), and by tenfold the risk for FM/CFS (OR = 0.09; 95% C.I. = 0.0057–1.65).

The (CCTTT)_9_ allele and the (CCTTT)_12_ allele had a lower and higher frequency, respectively, in FM/CFS patients than in all other groups that showed similar frequencies. The (CCTTT)_10_ allele had similar frequencies in all groups (Table 2).

The (CCTTT)_11_ allele frequency was around threefold higher in FM/CFS patients than in controls, and this difference was statistically significant (*P* < 0.01) (Table 2). Moreover, a difference tending to statistical significance was found when comparing FM/CFS patients with MCS (*P* = 0.06). This NOS2A variant was associated with a significantly increased risk for FM/CFS (OR = 3.557, 95% C.I. 1.575–8.033).

The (CCTTT)_13_ allele was more represented in MCS patients than in other groups and had a frequency almost double than in SMCS patients, but these differences were not statistically significant. The (CCTTT)_14_ and (CCTTT)_15_ alleles had similar frequencies in all groups.

The (CCTTT)_16_ allele was more represented in SMCS patients than in other groups, with a frequency that was eight- and twofold higher than in MCS patients and controls, respectively, but this difference was statistically significant (*P* < 0.01) only in comparison with MCS patients, while tended to be significant when compared with controls (*P* < 0.06). The lowest frequency was observed in MCS patients, with a significant difference in comparison with controls (*P* < 0.05).

The (CCTTT)_*n*_ genotype frequencies did not deviate from the expected value by Hardy-Weinberg equilibrium.

The (CCTTT)_8/13_ genotype was only found in controls, with a frequency of 4.5%.

The (CCTTT)_12_ allele was mostly represented in homozygous state in all examined groups. The (CCTTT)_12/12_ genotype was the most frequent in all examined group except in FM/CFS (16.9% MCS, 12.5% SMCS, 12% FM/CFS, and 16.9% controls). In this latter group, the most represented genotype was the (CCTTT)_10/12_ genotype that showed a significantly higher frequency when compared with MCS patients (20 versus 2.5%; *P* = 0.0044) and a borderline significant difference when compared with controls (20 versus 6.7%; *P* = 0.061), while frequencies in SMCS patients and controls were similar (6.3% versus 6.7%, *P* > 0.05).

The (CCTTT)_10/10_ genotype, having a frequency around 7% in MCS, SMCS, and controls, was not found in FM/CFS patients. The (CCTTT)_10/11_ in MCS patients had a frequency double than that in controls (10.2 versus 4.5%, not significant), while in SMCS patients and FM/CFS it accounted for 6.3% and 8.0%, respectively.

The (CCTTT)_11/11_ genotype had the highest frequency in FM/CFS patients (8% versus 3% in all other groups), but this difference did not reach statistically significant values.

The (CCTTT)_11/12_ genotype had the highest frequency in SMCS patients (12.5% versus 3.4% MCS versus 8% FM/CFS versus 3.4% controls), but this difference was not statistically significant.

The (CCTTT)_11/13_ genotype was more represented in FM/CFS patients than in other groups, with a frequency significantly different when compared with SMCS (16 versus 0%; *P* = 0.032) and controls (16 versus 1.1%;  *P* = 0.008) and borderline significant when compared with MCS (16 versus 4.2%; *P* = 0.0501). Moreover, this genotype was associated with an increased risk for FM/CFS (OR = 16.8; 95% C.I. = 1.78–157.9).

The (CCTTT)_13/13_ genotype had the highest frequency in MCS patients (6.8% versus 3.1% SMCS versus 0% FM/CFS versus 4.5% controls), but this difference was not statistically significant.

All other allele combinations did not reach a frequency higher than 5% in all groups.

In an attempt to better understand whether the presence of increasing −2.5 kb (CCTTT) repeat numbers of NOS2A promoter may be a genetic feature useful to discriminate different types of IEI, we grouped together different genotypes by classifying alleles with repeat number <12 as short (s) and alleles with repeat number ≥12 as long (l). The between-groups comparison of s and l allele frequencies showed that there were no significant differences between different groups of patients and also between patients and controls.

The heterozygous genotype (CCTTT)_short_/(CCTTT)_long_ (sl) showed the highest frequencies in FM/CFS and SMCS patients that were significantly different in comparison with both controls (*P* < 0.001, *P* = 0.02, resp.) and MCS patients (*P* < 0.001, *P* = 0.005, resp.); additionally, SMCS patients having the (CCTTT)_sl_ genotype were found to be significantly different from FM/CFS (*P* = 0.0016). Notably, this genotype increased by about fivefold and twofold, respectively, the risk for FM/CFS (OR = 4.8; C.I. = 2.813–8.211) and SMCS (OR = 1.9; C.I. = 1.129–3.048). Finally, the heterozygous genotype (CCTTT)_s_/(CCTTT)_l_ may be useful for diagnosis of either SMCS or FM/CFS states and discrimination between SMCS and either MCS or FM/CFS.

The (CCTTT)_long_/(CCTTT)_long_ (ll) genotype had the lowest frequency in FM/CFS patients and statistically significant differences were found in comparison with all other groups (MCS: *P* < 0.0001; SMCS: *P* = 0.04; controls: *P* = 0.0002). Therefore, the (CCTTT)_ll_ genotype may be useful to discriminate FM/CFS patients from both MCS and SMCS patients.

The (CCTTT)_short_/(CCTTT)_short_ (ss) genotype showed the lowest frequency in FM/CFS patients that were significantly different in comparison with controls (*P* = 0.012) and MCS patients (*P* = 0.015). Moreover, this genotype was protective against FM/CFS since it reduced by 2.5-fold the disease risk (OR = 0.4334; C.I. = 0.2269–0.8280).

### 3.3. Influence of NOS2A and NOS3 Polymorphisms on the Variability of Nitrite/Nitrate Plasma Levels

Analysis of nitrite/nitrate plasma concentrations showed that IEI patients recruited for this study exhibited similar levels of these proinflammatory markers (26.2 ± 13.3 *μ*mol/L MCS; 24.7 ± 12.2 SMCS; 28.4 ± 15.2 FM/CFS). Notably, nitrite/nitrate concentrations in patients were around or more than twofold higher than in controls (27.1 ± 13.8 versus 15.3 ± 5.2 *μ*mol/L), and this difference was statistically significant (*P* = 0.037).

**Figure 1:**
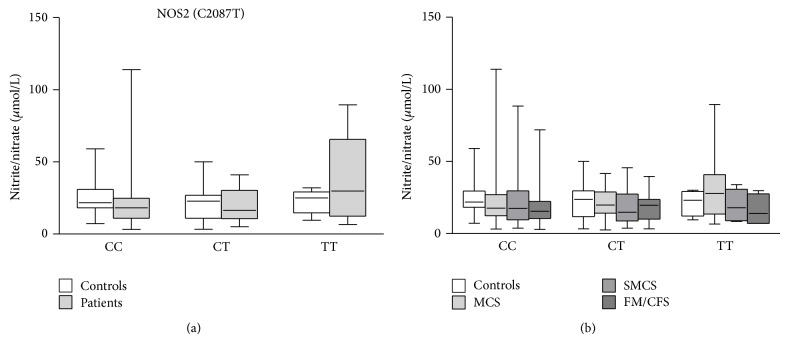
Effects of different NOS2A C2087T (Ser608Leu) genotypes on nitrite/nitrate plasma levels in IEI patients (MCS = 170; SMCS = 108; FM/CFS = 89), grouped together (a) or separated (b), and controls (*N* = 196). Box-plots graphically depict the variation in plasma nitrite/nitrate concentrations of patients and healthy subjects, having different NOS2A C2087T genotypes, through their quartiles. The bottom and top of the boxes are the first and third quartiles, and the band inside the boxes is the second quartile (the median); lines extending vertically from the boxes (*whiskers*) indicate variability outside the upper and lower quartiles (the ends of the whiskers represent the minimum and maximum of all of the data). MCS, multiple chemical sensitivity; SMCS, suspected multiple chemical sensitivity; FM/CFS, fibromyalgia/chronic fatigue syndrome; controls, healthy subjects.

We analyzed the variability of nitrite/nitrate concentrations in patients and controls having different NOS2A and NOS3 genotypes. Patients with different NOS2 C2087T genotypes showed different nitrite/nitrate levels, even if these differences did not reach statistical significance. In particular, TT homozygous mutated patients showed higher nitrite/nitrate concentrations than patients with other genotypes, while controls with different genotypes exhibited similar mean nitrite/nitrate plasma levels. TT homozygous patients had higher nitrite/nitrate levels compared with controls with the same genotype, while wild-type patients showed lower nitrite/nitrate levels than controls. However, no significant differences were observed when comparing patients and controls with different genotypes (Figure 1(a)). We tried to better investigate the between-groups differences, if present, by dividing IEI patients on the basis of their diagnosis. Again, no significant differences were found between different groups of patients and controls (Figure 1(b)).

**Figure 2:**
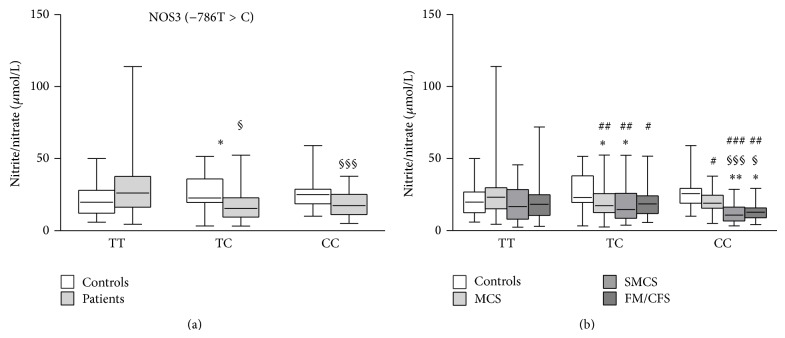
Effects of different NOS3 −786T>C genotypes on nitrite/nitrate plasma levels in IEI patients (MCS = 170; SMCS = 108; FM/CFS = 89), grouped together (a) or separated (b), and controls (*N* = 196). Box-plots graphically depict the variation in plasma nitrite/nitrate concentrations of patients and healthy subjects, having different NOS3 −786T>C genotypes, through their quartiles. The bottom and top of the boxes are the first and third quartiles, and the band inside the boxes is the second quartile (the median); lines extending vertically from the boxes (whiskers) indicate variability outside the upper and lower quartiles (the ends of the whiskers represent the minimum and maximum of all of the data). (a) ^*^ *P* < 0.05, significant difference in comparison with controls; ^§^ *P* < 0.05, ^§§§^ *P* < 0.001, significant difference in comparison with TT patients. (b) ^*^ *P* < 0.05, ^**^ *P* < 0.01 significant difference in comparison with controls; ^§^ *P* < 0.05, ^§§§^ *P* < 0.001 significant difference in comparison with TC controls; ^#^ *P* < 0.05, ^##^ *P* < 0.01, ^###^ *P* < 0.001 significant difference in comparison with TT MCS.

When nitrite/nitrate levels were analyzed in patients and controls with different NOS3 −786T>C genotypes, some statistically significant differences were found. In particular, nitrite/nitrate levels were significantly higher in TT wild-type patients than in either TC heterozygous or CC mutated patients (*P* = 0.012, *P* = 0.0007, resp.). Moreover, when comparing controls and patients a statistically significant difference was observed between TC heterozygous subjects, since controls showed higher nitrite/nitrate levels than patients with the same genotype (Figure 2(a)). When patients were divided in subgroups on the basis of different diagnosis, significant differences were found between either MCS or SMCS patients with TC genotype and controls having the same genotype (*P* = 0.04, *P* = 0.03, resp.). Moreover, SMCS and FM/CFS patients bearing the CC genotype had significantly lower nitrite/nitrate plasma levels than either controls having the same genotype (*P* = 0.002, *P* = 0.03, resp.) or TC genotype (*P* = 0.0005, *P* = 0.014, resp.) (Figure 2(b)). Finally, MCS patients having the TT genotype had nitrite/nitrate levels significantly higher than either SMCS or FM/CFS patients with TC (*P* = 0.008, *P* = 0.04, resp.) and CC genotype (*P* = 0.0001, *P* = 0.004, resp.) and MCS patients having either TC or CC genotype (*P* = 0.004, *P* = 0.03, resp.) (Figure 2(b)).

**Figure 3:**
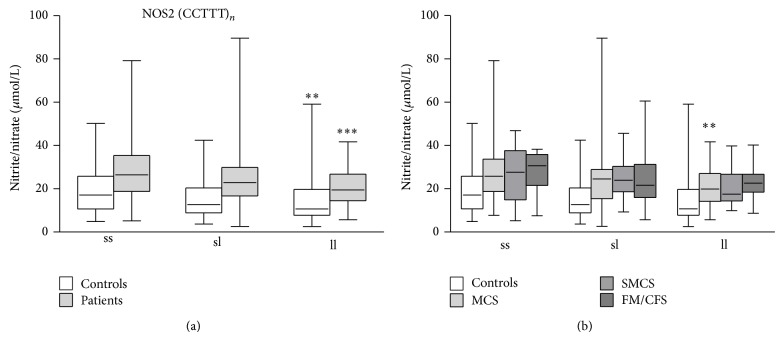
Effects of different NOS2 −2.5 kb (CCTTT)_*n*_ short (s) versus long (l) repeats alleles on nitrite/nitrate plasma levels in IEI patients (MCS = 170; SMCS = 108; FM/CFS = 89), grouped together (a) or separated (b), and controls (*N* = 196). Box-plots graphically depict the variation in plasma nitrite/nitrate concentrations of patients and healthy subjects, having different NOS2 −2.5 kb (CCTTT)_*n*_ genotypes, through their quartiles. The bottom and top of the boxes are the first and third quartiles, and the band inside the boxes is the second quartile (the median); lines extending vertically from the boxes (whiskers) indicate variability outside the upper and lower quartiles (the ends of the whiskers represent the minimum and maximum of all of the data). (a) ^**^ *P* < 0.01, ^***^ *P* < 0.001 significant difference versus subjects with (CCTTT)_s_/(CCTTT)_s_ genotype; (b) ^**^ *P* < 0.01, significant difference versus MCS with (CCTTT)_s_/(CCTTT)_s_ genotype.

Unfortunately, we could not analyze in detail the influence of the NOS2A −2.5 kb (CCTTT)_*n*_ pentanucleotide microsatellite on the variation of nitrite/nitrate plasma levels due to the dramatically high fragmentation of our study cohort caused by the high number of combinations of different NOS2A −2.5 kb (CCTTT) alleles. However, we tried to detect differences among patient subgroups by grouping (CCTTT) repeats as short (s) when <12 or long (l) when ≥12. By this way, we obtained three types of allele combinations, namely, (CCTTT)_s_/(CCTTT)_s_, (CCTTT)_s_/(CCTTT)_l_, and (CCTTT)_l_/(CCTTT)_l_. Then, we compared patients, grouped together (Figure 3(a)) or separated according to their diagnosis (Figure 3(b)), with controls having the same allele combinations. The results showed that nitrite/nitrate plasma levels were significantly higher in all patients and controls having the (CCTTT)_s_/(CCTTT)_s_ allele combination compared with patients and controls having the (CCTTT)_l_/(CCTTT)_l_ allele combination (*P* = 0.0002, *P* = 0.004, resp.) (Figure 3(a)). Moreover, while examining the subgroups of patients significant differences were found between MCS patients having the (CCTTT)_s_/(CCTTT)_s_ allele combination and those with (CCTTT)_l_/(CCTTT)_l_ allele combination (*P* = 0.008) (Figure 3(b)). However, two-way ANOVA, making a between-groups comparison, showed that nitrite/nitrate plasma levels were neither significantly different in subgroups of patients having different (CCTTT)_s_ and (CCTTT)_l_ allele combinations nor significantly different in comparison with controls having the same allele combinations (Figure 3(b)).

## 4. Discussion

Environment-associated pathologies have been attracting growing attention in the recent years due to increasingly daily exposure to various hazardous stimuli, such as chemicals, drugs, metals, electromagnetic or nuclear radiations, iatrogenic factors (including synthetic implants), specific foods, and microbial and environmental allergens [11]. Ethical, medical, and social/occupational impact of IEI is worsened by steadily growing population of hypersensitive people, not yet regulated diagnostic protocols, and abuse of not evidence-based treatments.

The most important studies on hypersensitive populations, especially MCS patients, have been concentrated on the search of genetic determinants of abnormal sensitivity. Based on the primary hypothesis of an impairment in the metabolism of xenobiotics, a wide variety of polymorphisms of genes encoding for phases I and II of detoxification enzymes were studied, such as CYPs, GST, COMT, UGT, NAT, and PON, with conflicting results [6, 10, 24–26]. Our group recently demonstrated that gene variants of GST and CYP isoforms, namely, GSTP1, GSTM1, GSTT1, CYP2C9∗2, CYP2C9∗3, CYP2C19∗2, CYP2D6∗4, and CYP2D6∗41, could represent genetic determinants for IEI and hence may be used as markers for differential diagnosis of various IEI [7, 10]. The patients recruited for this study were diagnosed clinically by means of standard validated questionnaires (QEESI). Previous analyses of specific panels of genetic and metabolic markers identified as pathognomonic for the diseases [6, 7, 9–11] had confirmed the peculiar alterations of antioxidant enzyme activities (catalase, glutathione-S-transferase, and glutathione peroxidase), low cellular and plasmatic concentrations of antioxidant molecules (reduced glutathione, plasma total antioxidant activity), increased oxidative stress indices (4-hydroxy-2-nonenal, whole blood luminol-dependent chemiluminescence), increased plasma levels of inflammatory markers (cytokines), and altered concentrations of erythrocyte membrane fatty acid saturated, monounsaturated, and polyunsaturated and fatty acid omega-6 and omega-3 [9, 10]. Moreover, genetic analyses had shown that patient subgroups were bearing one or more mutated variants of detoxifying enzymes, namely, GSTP1, GSTM1, GSTT1, CYP2C9∗2, CYP2C9∗3, CYP2C19∗2, CYP2D6∗4, and CYP2D6∗41, proven to be useful for diagnostic assessment of IEI patients [6, 7, 10, 11].

A number of years ago, Pall and Satterlee [14] postulated the existence of a vicious nitric oxide/peroxynitrite cycle as the basis for IEI, starting from several observations: (1) several organic solvents, which induce chemical sensitization (formaldehyde, benzene, carbon tetrachloride, and certain organochlorine pesticides), trigger increases in nitric oxide levels; (2) organophosphate and carbamate insecticides are suggested to induce MCS by inactivating acetylcholinesterase and thus activating muscarinic response, which is known to produce increases in nitric oxide; (3) elevated cytokines, an integral part of the proposed feedback mechanism of the elevated nitric oxide/peroxynitrite theory, are known to be induced by organic solvents through NOS2 activation; (4) antioxidant therapy has been reported to improve IEI symptoms, as expected if the levels of oxidant peroxynitrite are elevated; (5) the symptoms exacerbated after chemical exposure may be explained by several known proinflammatory properties of nitric oxide, peroxynitrite, and inflammatory cytokines, each of which have a role in the proposed mechanism; and (6) IEI are often treated through intramuscular injections of vitamin B12, that, in the form of hydroxocobalamin, is a potent nitric oxide scavenger in vitro and in vivo.

Lately, it has been reported that nitrite/nitrate levels, responsible for lipid peroxidation and cytokine increase, are significantly increased in IEI patients [11]. Interestingly, circulating NO and consequently nitrite/nitrate levels may be altered by the presence of NOS polymorphisms [21, 27, 28]. Moreover, some NOS variants, particularly the NOS2 Ser608Leu as well as the pentanucleotide microsatellite −2.5 kb (CCTTT)_*n*_ and the NOS3 −786T>C, were associated with either increased nitrite/nitrate levels, total serum immunoglobulin E, and blood eosinophil levels or cytokines in various allergic/inflammatory/autoimmune disorders, including atopy, asthma, migraine, inflammatory bowel disease, or rheumatoid arthritis [16–20, 29–33].

Given that allergic/inflammatory symptoms are common in IEI patients and that our patients were all exhibiting remarkably elevated nitrite/nitrate plasma levels compared to controls (27.1 ± 13.8 versus 15.3 ± 5.2 *μ*M, *P* < 0.05), we chose to investigate the distribution of the polymorphic variants NOS2 −2.5 kb (CCTTT)_*n*_ and Ser608Leu and NOS3 −786T>C in IEI patients.

We did not find significant difference in the distribution of the polymorphic variants NOS2A C2087T (Ser608Leu) and NOS3 −786T>C when patient groups were compared with controls (Table 1). Allele and genotype frequencies in healthy subjects were similar to those reported in other European populations [29]. Moreover, while investigating the effects of these NOS2A and NOS3 variants on the variability of nitrite/nitrate plasma levels, we could not observe any effect of NOS2A C2087T. Unfortunately, no information is available on the influence of NOS C2087T on variation of these inflammatory markers. So, this is the first report documenting no apparent effect of the NOS2A Ser608Leu on the variation of nitrite/nitrate plasma levels.

Interestingly, we found that patients with the NOS3 −786TT wild-type genotype had significantly increased nitrite/nitrate levels in comparison with patients having other genotypes. This latter observation agrees with those previously reported in patients with coronary spastic angina and healthy subjects after exercise [21, 28] and is in contrast with others in patients with asthma [27]. We also tried to detect differences in nitrite/nitrate plasma levels between subgroups of patients having different genotypes. What we found is that the significant difference initially observed between plasma levels of nitrite/nitrate in TT patients and those of either TC or CC patients (Figure 2(a)) was largely attributable to MCS patients bearing the TT genotype that had nitrite/nitrate concentrations significantly higher than MCS, SMCS, and FM/CFS patients bearing either TC of CC genotype.

Notably, we detected a relationship between the −2.5 kb (CCTTT)_*n*_ microsatellite in the promoter region of NOS2A and IEI (Table 2). The NOS2A −2.5 kb (CCTTT)_*n*_ microsatellite has been so far associated with asthma, atopy, rheumatoid arthritis, and inflammatory bowel disease [17, 18, 29–31]. It represents an attractive disease-causing candidate for IEI, being a highly polymorphic marker (11 different alleles, ranging 7–17 repeats, and 171–221 bp) with a suggested effect in NOS2 transcription. Indeed, it has been reported that variations in the number of (CCTTT) repeats are functionally important in the regulation of NOS2 transcription, leading to increase in NO production [34].

Highly significant differences have been reported in the (CCTTT) allele frequencies between ethnically diverse populations [35]. The observed frequencies of this marker in our cohort of healthy subjects fell within the Caucasian pattern, with the peak at the 12-repeat allele [29, 31, 35, 36]. When we compared the distribution of the (CCCTT) repeat within the control subjects with that of IEI patients, the 8-repeat allele showed a significantly decreased frequency in the groups of patients compared with controls (Table 2). The odds ratio calculation showed that the presence of this allele reduced the risk for the different types of IEI examined in this study, suggesting that this NOS2A variant may display a protective effect against these disorders.

On the contrary, the 11-repeat allele, showing a significantly increased frequency in the group of FM/CFS patients compared with controls, may be considered a genetic determinant for FM/CFS. Moreover, the (CCTTT)_16_ allele, having a significantly different frequency between MCS and SMCS, may be used to discriminate these two types of IEI.

Moreover, taking into account different repeat cut offs (9, 11, 13, and 16 repeats), we found that a different number of NOS2A (CCTTT) repeats could be used to discriminate between the different pathological conditions. Interestingly, when we grouped the (CCTTT)_*n*_ alleles in two categories according to the number of repeats, that is, the alleles with <12 repeats were designated as s (short) and alleles with ≥12 repeats as l (long) alleles, as previously described [29], we found that the (CCTTT)_s_/(CCTTT)_l_ allele combination had the highest frequencies in FM/CFS and SMCS (Table 3). Therefore, this NOS2A allele combination may be useful for diagnosis of these two types of IEI. Moreover, the observed distribution of (CCTTT)_s_/(CCTTT)_l_ and (CCTTT)_l_/(CCTTT)_l_ allele combination (Table 3) confirms that SMCS patients had overlapping features with FM/CFS and MCS and may be considered as an intermediate pathological condition between the two above cited.

After grouping of different (CCTTT) allele combinations, we were able to investigate the relationship between this polymorphic variant and nitrite/nitrate plasma levels in patients. Thus, we found that a short number of (CCTTT) repeats allele was associated with higher concentrations of nitrites/nitrates.

## 5. Conclusions

Our results demonstrated for the first time that the NOS2A promoter pentanucleotide microsatellite −2.5 kb (CCTTT)_*n*_ is associated with FM/CFS and may be feasible for the diagnostic assessment of this type of IEI. Moreover, the screening for the presence of some NOS2A −2.5 kb (CCTTT) variants, that is, the 8- and 16-repeat alleles, may be useful, respectively, to exclude the diagnosis of IEI and discriminate between MCS and SMCS.

## Figures and Tables

**Table 1:** Allele and genotype frequencies of NOS2A and NOS3 SNPs in IEI patients and healthy subjects.

Genotype	MCS (*N* = 170)	SMCS (*N* = 108)	FM/CFS (*N* = 89)	Controls (*N* = 196)
**NOS2A ** **C2087T (Ser608Leu)**				
CC (Ser/Ser)	60.6%	52.8%	65.2%	60.2%
CT (Ser/Leu)	34.7%	41.7%	30.3%	36.2%
TT (Leu/Leu)	4.7%	5.6%	4.5%	3.6%
C allele frequency	0.78	0.74	0.80	0.78
T allele frequency	0.22	0.26	0.20	0.22
−**786T>C NOS3**				
TT	32.9%	34.3%	33.7%	33.2%
TC	45.9%	46.3%	49.4%	44.5%
CC	21.2%	19.4%	16.9%	22.3%
T allele frequency	0.56	0.56	0.58	0.55
C allele frequency	0.44	0.44	0.42	0.45

MCS, multiple chemical sensitivity; SMCS, suspected multiple chemical sensitivity; FM/CFS, fibromyalgia/chronic fatigue syndrome; controls, healthy subjects.

**Table 2:** Allelic distributions of the NOS2A promoter pentanucleotide microsatellite −2.5 kb (CCTTT)_*n*_ in IEI patients and healthy subjects.

Genotype NOS2A −2.5 kb (CCTTT)_*n*_	MCS (*N* = 170)	SMCS (*N* = 108)	FM/CFS (*N* = 89)	Controls (*N* = 196)
8	0.03^a^	0.02^b^	0^c^	0.09
9	0.07	0.09	0.04	0.08
10	0.17	0.19	0.18	0.18
11	0.15	0.16	0.26^d^	0.09
12	0.28	0.27	0.32	0.27
13	0.15	0.08	0.10	0.12
14	0.07	0.05	0.04	0.06
15	0.07	0.08	0.04	0.07
16	0.01^e,f^	0.08	0.02	0.04

MCS, multiple chemical sensitivity; SMCS: suspected multiple chemical sensitivity; FM/CFS, fibromyalgia/chronic fatigue syndrome; controls, healthy subjects. a: *P* = 0.0094, significant difference in comparison with controls; b: 0.0482, significant difference versus controls; c: *P* = 0.026, significant difference versus controls; d: *P* = 0.0031, significant difference versus controls; e: *P* = 0.0426, significant difference versus controls; f: *P* = 0.0057, significant difference versus SMCS.

**Table 3:** Genotype and allele frequencies of the NOS2A promoter pentanucleotide microsatellite −2.5 kb (CCTTT)_*n*_ short and long repeats in IEI patients and healthy subjects.

Genotype	MCS (*n* = 170)	SMCS (*n* = 108)	FM/CFS (*n* = 89)	Controls (*n* = 196)
(CCTTT)_s_/(CCTTT)_s_	29.4%^§^	25.0%	15.7%^*^	30.1%
(CCTTT)_s_/(CCTTT)_l_	24.7%^##,§§§^	40.7%^∗,§§^	64.1%^***^	27.0%
(CCTTT)_l_ /(CCTTT)_l_	45.9%^§§§^	34.3%^§^	20.2%^***^	42.9%
Allele frequency				
(CCTTT)_s_	0.4	0.5	0.5	0.4
(CCTTT)_l_	0.6	0.5	0.5	0.6

MCS, multiple chemical sensitivity; SMCS: suspected multiple chemical sensitivity; FM/CFS, fibromyalgia/chronic fatigue syndrome; controls, healthy subjects. s: short repeats (CCTTT)_8–11_; l: long repeats (CCTTT)_12–16_. ^*^ *P* < 0.05, ^***^ *P* < 0.001 significant difference in comparison with controls. ^§^ *P* < 0.05, ^§§^ *P* < 0.01, and ^§§§^ *P* < 0.001 significant difference in comparison with FM/CFS. ^##^ *P* < 0.01 significant difference in comparison with SMCS.
